# Assessment of the Eppendorf ERIS analyser

**DOI:** 10.1155/S1463924685000207

**Published:** 1985

**Authors:** S. J. Callaghan, R. Singer, J. M. White, C. G. Fraser

**Affiliations:** Department of Biochemical Medicine Ninewells Hospital and Medical School Dundee DD1 9SY UK


**Journal of Automatic Chemistry**/*Journal of Clinical Laboratory Automation*, **Vol. 7, No. 2 (Apr–Jun 1985), pp. 90–94**

### Assessment of the Eppendorf ERIS analyser

**S. J. Callaghan, R. Singer, J. M. White and C. G. Fraser***

*Department of Biochemical Medicine, Ninewells Hospital and Medical School, Dundee DD1 9SY, UK*

### Introduction

The Eppendorf ERIS, a selective multi-test analyser, capable of performing up to 20 tests on each sample, was evaluated in order to assess its suitability as at least a partial replacement for the parallel fast analysers currently used at Ninewells Hospital [1]. The assessment was carried out following the protocols delineated by the Expert Panel on Principles and Nomenclature of Quality Control in Clinical Chemistry of the International Federation of Clinical Chemistry [2] and by Broughton *et al.* [3]. In order to assess different types of assay, the performance characteristics of glucose, cholesterol, triglycerides, iron, urate, aspartate aminotransferase (AST; EC 2.6.1.1.) and γ-glutamyl transferase (GGT; EC 2.3.2.2.) analyses were evaluated.

### Materials and methods

*The instrument*

The Eppendorf ERIS originated from the Olympus Optical Company Ltd of Japan, but Eppendorf (Eppendorf Geratabau Netheler and Hinz GmbH, FR Germany) have considerably modified the instrument with new data-handling software and a dispensing system of their design. The instrument was kindly provided by BDH Diagnostics (Poole, UK) for the assessment.

The instrument consists of two modules: an analyser module, which is free-standing, measures 101 × 74 × 109 cm and weighs approximately 350 kg; and a free-standing data processor, 62 × 76 × 109 cm. A high-speed printer in an integral part of the system.

The analyser module has a central reaction rotor maintained at 37° ± 0.1 °C by a closed water system. The rotor has 72 spaces for disposable plastic reaction cuvettes of 1 cm path-length. The cuvettes, which are prepacked in cassettes of 100 and up to 10 of which may be stored in the analyser, are loaded and unloaded automatically into the rotor which advances one position every 9 s. The cuvettes are prewarmed to 37 °C before loading and are unloaded into a plastic disposable bag. At the centre of the rotor is the 12 V 100 W halogen lamp. Wavelength selection is performed by an interference filter wheel between lamp and cuvettes. There are 15 photometers around the rotor, one of which is used to check cuvette acceptability and to perform reagent blank absorbance measurements; the other 14 photometers are used in assay absorbance measurements. All assays are biochromatic, reducing the requirement for assay of sample blanks.

The samples are introduced in cups of 1.4 ml capacity using a plastic inter-locking chain-link system. Up to 100 samples may be maintained in the high humidity sample chamber. Sample identification is serial and standards, quality controls and patient specimens are recognized by bar codes on the links of the chain. Emergency samples may be introduced using a dedicated facility at the dispensing station, and a sequence of urgent tests may be simply performed by insertion of coded links in the sample chain at any point.

Reagents, in 18 ml bottles sufficient for 180–720 analyses (depending on methodology), are of concentrated form and are prepared by E. Merck (Darmstadt, FR Germany). They are stored in a refrigerated (8 °C) carousel and an automatic uncapping and recapping system minimizes evaporation.

Samples and reagents are dispensed using precision stepper motor pumps with magnetically controlled valves. One pump is for sample dispensing and sample probe rinsing, two for reagent dispensing and reagent probe rinsing, and five for washing probes and stirrers. The dispensing pumps are controlled by three arms situated between the sample station and the rotor. Sample volume is in 1 μl steps from 5–25 μl and transfer of samples occurs after reagent transfer; the probes are rinsed automatically with distilled water. Reagent volumes are adjustable from 25–100 μl in 5 μl steps; up to two reagents may be simultaneously added. All reagents are concentrated and a four-fold volume of distilled water is dispensed simultaneously from a 10 l reservoir. Reagent probes are washed following dispensing of reagent. Sample and reagent probes possess liquid level detectors which are self-adjusting after each aspiration.

The analyser produces one result every 9 s after a lag period of 10 min, so a maximum throughput of 400 tests/h is possible.

Calibration is generally one-point and materials and levels may be selected by the user. The manufacturer recommends that calibration be performed at weekly intervals or when changes of reagents or other parameters occur.

The data-processing module is programmable and has three types of floppy disk. Program disks are required to store routine and laboratory-defined method parameters, which are loaded each day. Data disks store patient demographic data and results. Quality control disks store data for up to 31 days and provide statistical and graphical analyses. Test selection is performed by 'yes/no' keyed responses to computer prompts on a test menu

* *Corresponding author.*

displayed on the VDU screen. Patient identification and data is entered by means of a keyboard. Data can be displayed on the screen, the integral printer or a high-speed printer. When the high-speed printer is used, report forms specific to the requirements of the laboratory may be used.

### Analytical methods and reagents

All reagents for use on the ERIS were supplied by E. Merck. Analytical details of the methods are shown in table 1; all methods were run at 37 °C.

The comparison methods were performed on a Rotochem IIa parallel fast analyser (Aminco, Silver Spring, Massachusetts, USA). Analytical methods were hexokinase/dehydrogenase for glucose using glucose-HK endpoint reagent (Beckman Dri-STAT, Beckman Instruments Inc., Carlsbad, California, USA), cholesterol oxidase/4-aminophenazone colorimetry for cholesterol using the C-system of Boehringer Mannheim GmbH (Mannheim, FR Germany), chymotrypsin/lipase/glycerol kinase/pyruvate kinase/lactate dehydrogenase for triglyceride using SKI reagents (Smith Kline Instruments, Vienna, Austria); tripyridyl-s-triazine/thioglycollic acid for iron using in-house reagents; uricase/catalase/aldehyde dehydrogenase for urate using SKI reagents, the malate dehydrogenase/$NADH^+$ reaction for AST using SKI reagents and hydrolyses of γ-glutamyl *p*-nitroanilide for GGT using SKI reagents. All reactions were end-point, except the two enzyme activity assays.

Control sera used were Wellcome 1, 2 and Autoset High (Wellcome Diagnostics, Dartford, Kent, UK), Precipath U and Precinorm U (Boehringer Mannheim GmbH), Target Normal and Target Abnormal (Smith Kline Instruments), Versatol Lo and Hi (General Diagnostics, Morris Plains, New Jersey, USA) and five different materials from Wellcome Diagnostics which the laboratory had analysed in replicate in order to allow the manufacturer to assign consensus values to the material.

Calibration was performed in essentially the same way on the Eppendorf ERIS and the comparison methods using Autoset High (Wellcome Diagnostics) for glucose, Precilip E. L. (Boehringer Mannheim GmbH) for cholesterol, an in-house glycerol standard for triglycerides, the ERIS calibrator (E. Merck, Darmstadt, FR Germany) for iron and the Autoset High for urate analyses.

### *Sample evaporation*

Twenty sample cups were filled with 500 μl of a control serum and these were analysed seven times over a 3 h period at 30 min intervals.

### *Cuvette integrity*

In order to detect any variation in cuvette path-length, the absorbance of a standard solution of potassium dichromate in 5.0 mmol/l sulphuric acid (Spectrasol, BDH, Poole, UK) was measured at 340 nm in 50 different cuvettes.

### *Photometer equivalence*

The absorbance of the potassium dichromate in 5.0 mmol/l sulphuric acid was measured in each of the 14 photometers.

### *Carry-over*

Total carry-over was assessed using the formula of Broughton *et al.* [3]. The high and low sera had glucose, cholesterol, triglycerides, iron, urate, AST and GGT levels of 16.9 mmol/l, 9.73 mmol/l, 3.56 mmol/l, 57.1 μmol/l, 0.67 mmol/l, 260 U/l and 579 U/l, and 2.0 mmol/l, 2.11 mmol/l, 0.50 mmol/l, 6.3 μmol/l, 0.25 mmol/l, 17 U/l and 12 U/l respectively.

### *Imprecision*

Control sera at three different levels of analyte were assayed approximately 30 times in a single batch in order to assess within-run imprecision, and were assayed in 28 separate analytical batches to evaluate between-run imprecision.

### *Relative inaccuracy*

Approximately 100 sera from patients were analysed on the Eppendorf ERIS and by the comparison methods detailed earlier. The sera were carefully selected to cover the analytical range of the methods and were also selected to represent typical specimens presented to the laboratoy for analysis with respect to icterus, lipaemia and hae-

*Table 1. Details of methods used on the Eppendorf ERIS.*

| Analyte | Method principle | Sample volume (μl) | Reagent volume (μl) | Peak λ (nm) | Side band λ (nm) | Assay technique |
|---|---|---|---|---|---|---|
| Glucose | Glucose dehydrogenase | 5 | 100 | 340 | 380 | Fixed time |
| Cholesterol | Cholesterol oxidase/ formation of quinonimine | 7 | 100 | 540 | 600 | Fixed time |
| Triglycerides | Lipase/glycerol kinase/ glycerol phosphate oxidase/ peroxidase | 5 | 95 | 520 | 600 | End point |
| Iron | Tripyridyl-s-triazine | 25 | 50 | 600 | 800 | Fixed time |
| Urate | Uricase/peroxidase | 5 | 55 | 520 | 600 | Fixed time |
| AST | Malate dehydrogenase/$NADH^+$ SCE recommendations | 25 | 25 | 340 | 380 | Kinetic |
| GGT | 5-amino-2-nitrobenzoate | 25 | 50 | 410 | 480 | Kinetic |

molysis in order to assess possible *interference*. The results were assessed graphically to give data on method *linearity*.

*Inaccuracy*

Twelve control sera of different matrix types and from a variety of commercial sources were each assayed 20 times and the mean result compared to the assigned or consensus value.

### Results and discussion

*Sample evaporation*

The means of the seven sets of analyses carried out over the 3 h period were not significantly different; it was therefore concluded that significant evaporation does not occur for at least this time period ($0.001 > p > 0.01$).

*Cuvette integrity*

The coefficient of variation of 50 analyses in different cuvettes was 0.28%. This is well within the cuvette path-length variation specification of 0.5% by the manufacturer.

*Photometer equivalence*

The coefficient of variation between the 14 photometers was 0.08%. The standard solution had an assigned absorbance of 0.5040 units and the mean reading achieved from the 14 photometers was 0.5036 units. In the absence of objective analytical goals for this parameter, it is empirically considered that these results are satisfactory since the photometric imprecision and inaccuracy would not contribute significantly to methodological imprecision and bias.

*Carry-over*

No carry-over was detected for glucose, cholesterol, triglycerides, AST and GGT analyses. Carry-over was 0.41% for iron, which is acceptable according to the criterion of Broughton *et al.* [2] that carry-over should be less than 2.0%. Carry-over for urate was found to be 2.4%, but, because of the imprecision at the low level of urate in the samples used in this study, it is considered that the apparent carry-over of 0.01 mmol/l is acceptable and could well have been due to random error.

*Imprecision*

Within-run imprecisions are shown in table 2 and between-run imprecisions are shown in table 3. The between-run imprecisions were generally significantly larger than the within-run imprecisions (F-test). Although there have been many criteria proposed for judging whether analytical imprecision is acceptable or not, the current expert consensus view [4] is that acceptable performance standards are those based upon intra-individual biological variation data [5]. Using this criterion, analytical goals for glucose, cholesterol, triglycerides, iron, urate, AST and GGT are, expressed as coefficients of variation, 2·2%, 2·4%, 13·0%, 13·0%, 3·7%, 7·5% and 11·6% respectively. All coefficients of variation found, with the exception of glucose at a level of 4.9 mmol/l – well within the reference range, were therefore acceptable for clinical purposes.

*Relative inaccuracy*

Relative inaccuracy was assessed using the criteria of Westgard and Hunt [6] by the simple linear regression method which assumes that the comparison methods are free from error. Statistical data is shown in table 4.

*Table 2. Within-run imprecisions at three levels of analyte.*

| Analyte | Units | *N* | Mean | SD | CV(%) |
|---|---|---|---|---|---|
| | | 30 | 5.0 | 0·05 | 1·0 |
| Glucose | mmol/l | 30 | 8·5 | 0·10 | 1·2 |
| | | 30 | 12·1 | 0·12 | 1.0 |
| | | 30 | 2·68 | 0·022 | 0·8 |
| Cholesterol | mmol/l | 29 | 6·23 | 0·042 | 0·7 |
| | | 30 | 9·82 | 0·078 | 0·8 |
| | | 30 | 0.66 | 0·012 | 1·8 |
| Triglycerides | mmol/l | 30 | 2·22 | 0·021 | 0·9 |
| | | 30 | 3·83 | 0·063 | 1·6 |
| | | 30 | 22·9 | 0·36 | 1·6 |
| Iron | μmol/l | 30 | 32·8 | 0·55 | 1·7 |
| | | 28 | 43·5 | 1·06 | 2·4 |
| | | 30 | 0·26 | 0·003 | 1·2 |
| Urate | mmol/l | 30 | 0·43 | 0·007 | 1·7 |
| | | 30 | 0·59 | 0·007 | 1·2 |
| | | 28 | 25 | 0·5 | 1·9 |
| AST | U/l | 30 | 77 | 0·7 | 0·9 |
| | | 29 | 133 | 0·7 | 0·6 |
| | | 30 | 42 | 0·5 | 1·2 |
| GGT | U/l | 30 | 85 | 0·6 | 0·7 |
| | | 30 | 128 | 0·8 | 0·6 |

*Table 3. Between-run imprecisions at three levels of analyte.*

| Analyte | Units | *N* | Mean | SD | CV(%) |
|---|---|---|---|---|---|
| | | 28 | 4.9 | 0·12 | 2·4 |
| Glucose | mmol/l | 28 | 8·4 | 0·17 | 2·1 |
| | | 28 | 11·9 | 0·20 | 1·7 |
| | | 28 | 2·66 | 0·040 | 1·5 |
| Cholesterol | mmol/l | 28 | 6·14 | 0·059 | 1·0 |
| | | 28 | 9·61 | 0·099 | 1·0 |
| | | 28 | 0·72 | 0·035 | 4·9 |
| Triglycerides | mmol/l | 28 | 2·16 | 0·043 | 2·0 |
| | | 28 | 3·65 | 0·095 | 2·6 |
| | | 28 | 22·6 | 0·47 | 2·1 |
| Iron | μmol/l | 28 | 34·0 | 0·82 | 2·4 |
| | | 28 | 44·4 | 0·70 | 1·6 |
| | | 28 | 0·27 | 0·011 | 4·1 |
| Urate | mmol/l | 28 | 0·43 | 0·011 | 2·6 |
| | | 28 | 0·60 | 0·013 | 2·1 |
| | | 28 | 23 | 0·7 | 2·0 |
| AST | U/l | 28 | 76 | 1·2 | 1·6 |
| | | 28 | 125 | 2·2 | 1·7 |
| | | 28 | 41 | 0·6 | 1·4 |
| GGT | U/l | 28 | 84 | 1·4 | 1·6 |
| | | 28 | 126 | 1·4 | 1·1 |

*Table 4. Data from regression analysis; y = Eppendorf ERIS; x = comparison method.*

| Analyte | No. of pairs | Slope | Intercept | Correlation Coefficient | $S_{y.x}$ | Mean $y$ | Mean $x$ | Student's t-value |
|---|---|---|---|---|---|---|---|---|
| Glucose | 100 | 0·99 | +0·08 | 0·998 | 0·031 | 8·0 | 8·1 | 1·40 |
| Cholesterol | 102 | 0·96 | +0·21 | 0·996 | 0·015 | 5·71 | 5·72 | 1·18 |
| Triglycerides | 102 | 0·96 | +0·07 | 0·995 | 0·011 | 1·57 | 1·57 | 0·19 |
| Iron | 95 | 1·04 | +0·81 | 0·990 | 0·179 | 12·4 | 11·1 | 6·83 |
| Urate | 102 | 1·01 | 0·00 | 0·979 | 0·003 | 0·38 | 0·38 | 1·23 |
| AST | 100 | 1·02 | −1·65 | 0·999 | 0·372 | 67 | 67 | 0·70 |
| GGT | 100 | 1·06 | +6·85 | 0·990 | 1·284 | 105 | 92 | 9·90 |

Graphical display of results showed that the glucose, cholesterol, triglycerides, iron, urate, AST and GGT methods were linear to at least 25·0 mmol/l, 9.00 mmol/l, 4·50 mmol/l, 50·0 µmol/l, 0·90 mmol/l, 425 U/l and 800 U/l respectively. There are very few goals available to assess the acceptability of these results for linearity; the methods are considered satisfactory and, for at least glucose and cholesterol, published goals [7] are fulfilled.

There were no gross outliers among the results for all methods, and it is concluded that interferences with the methods do not pose a significant problem. Statistically, correlations between the results obtained by the Eppendorf ERIS and the comparison methods were excellent, all being greater than 0·979. Neither significant systematic or proportional errors existed for glucose, cholesterol, triglycerides, urate and AST analyses and, since the comparison methods are, in our laboratory, enrolled in a multiplicity of quality assurance schemes which show that these methods have little bias, it is concluded that the results for the Eppendorf ERIS methods approach the goal of having negligible inaccuracy.

For iron analyses, the Eppendorf ERIS gave results which were significantly higher (t-test), due mainly to positive proportional bias; it is considered that the Eppendorf ERIS method did in fact have little inaccuracy since the comparison method has a small and consistent negative bias in quality assurance survey results.

The positive proportional bias in GGT results which is statistically significant (t-test) is attributed to the differences in substrate used on the Eppendorf ERIS (γ-glutamyl-5-amino-nitrobenzoate) and the comparison method (γ-glutamyl-*p*-nitroanilide).

*Inaccuracy*

The results obtained for analysis of the control sera fell within the stated limits of the materials, indicating that significant bias did not exist.

### Subjective assessment

The analyser cost £69 500 (price correct at November 1984) including a 12-month warranty, service contract of parts, labour and service engineer travel costs for 12 months, one training course in Poole, UK and further training during commissioning in the laboratory. The fixed costs of a yearly service contract are £5907 which adds 1·7 pence per test to the average reagent costs per test. If components for calibration, sample cups, cuvettes, paper, water and power are included, the total cost is 11·1 pence per test, based upon an average of six tests being done on 200 samples each day of 250 working days per annum.

The Eppendorf ERIS was reliable and required little routine maintenance, which consists only of daily inspection of alignment of probes, checking for leakage and general cleaning procedures. Only one minor fault, a stirrer paddle retaining spring breakage, occurred during the evaluation. A temporary repair was satisfactory and minimal down time occurred.

### Conclusions

The Eppendorf ERIS proved to be a reliable instrument which had acceptable performance characteristics for the analytes studied, having negligible carry-over, low imprecision, good analytical ranges and insignificant bias.

Extensive studies on linearity, interfering substances and recovery were not carried out due to time constraints but it is not likely that significant additional data would have been generated from such experiments.

The instrument, being able to analyse up to 20 tests per sample, a test result being available every 9 s after an initial lag period of 10 min, and being able to perform emergency tests during an analytical run, would be very suitable for small- to medium-size laboratories as the mainstay of the service. In a larger laboratory, it could very conveniently be used to analyse large batches of specimens for few analytes.

Many desirable features are present: simplicity of operation, small sample size, user-friendly data handling, availability of quality control data handling, liquid level sensors for sample and reagents, stability of reagents, large number (180–720) of tests per vial of concentrated reagent, refrigeration of reagents, negligible specimen evaporation and good report production, particularly using the high-speed printer.

### Acknowledgements

The authors thank BDH Diagnostics and their staff for the loan of the instrument and the provision of reagents and consumables; Mr P. Woodford and Ms A. Price were particularly helpful.

### References

1. Griffiths, P. D., Martin, B. T. and Cannon, G., in *Centrifugal Analysers in Clinical Chemistry*, Ed. Price, C. P. and Spencer, K. (Praeger Publishers, 1980), 83.
2. Buttner, J., Borth, R., Boutwell, J. H., Broughton, P. M. G. and Bowyer, R. C., *Clinica Chimica Acta*, **98** (1979), 145F.
3. Broughton, P. M. G., Gowenlock, A. H., McCormack, J. J. and Neill, D. W., *Annals of Clinical Biochemistry*, **11** (1974), 207.
4. Fraser, C. G., *Advances in Clinical Chemistry*, **23** (1983), 299.
5. Ross, J. W., in *CRC Handbook of Clinical Chemistry, Volume 1*, Ed. Werner, M. (CRC Press, 1982), 391.
6. Westgard, J. O. and Hunt, M. R. *Clinical Chemistry*, **19** (1973), 49.
7. Elevitch, F. R. *Proceedings of the 1976 Aspen Conference on Analytical Goals in Clinical Chemistry* (CAP, 1977), 4.

### FLOW ANALYSIS III

The Third International Conference on Flow Analysis will be held in Birmingham, UK from 5 to 8 September 1985, organized by the Midlands Region of the Analytical Division of the Royal Society of Chemistry. The scope of the conference will be similar to the previous Flow Analysis Meetings (in Amsterdam in 1979 and in Lund in 1982) and current research on all aspects of continuous flow analysis will be covered, including:

Instrumentation for flow injection analysis and for continuous segmented and unsegmented flow analysis, and approaches to total automation; New detector systems and hybrid systems; Theory of flow analysis; Applications in industrial, environmental and clinical analysis.

Invited lecturers include Dr Stewart, Professors Riley and Ruzička. Papers for inclusion can be submitted up to 15 April to Dr A. M. G. Macdonald, Department of Chemistry, The University, PO Box 363, Birmingham B15 2TT, UK.

*Registration forms are available now from The Secretary, Analytical Division, Royal Society of Chemistry, Burlington House, London W1V 0BN.*

### INFRA-RED SUMMER SCHOOL AT CAMBRIDGE

A five-day residential school at Cambridge, aimed at providing participants with a comprehensive understanding of the theory and the application of infra-red spectrophotometry, has been organized by Pye Unicam.

To take place from 29 July to 2 August 1985, with accommodation at Queen's College, the course has been designed to provide chemists with instruction in modern instrumental techniques, including data processing, spectral interpretation and the latest sample handling methods. Practical work will be carried out at Pye Unicam.

A sound organic chemistry background is required, together with an understanding of infra-red spectroscopy, for participants to gain maximum benefit. Experts will be on hand to advise on specific sampling, interpretation and application problems.

Guest speakers will include: Dr A. J. Baker, of the University of Glasgow, Dr D. B. Powell, University of East Anglia, Dr C. J. Timmons, University of Nottingham, and Mr P. Carter of FBC Ltd.

*A fee of £425 plus VAT includes five nights' accommodation, meals, tuition and course material. For further information please contact Mrs R. Fullerton at Pye Unicam, York Street, Cambridge CB1 2P4, UK, telephone 0223 358866, ext. 501.*

